# Probiotic Mixtures Consisting of Representatives of Bacteroidetes and Selenomonadales Increase Resistance of Newly Hatched Chicks to *Salmonella* Enteritidis Infection

**DOI:** 10.3390/microorganisms12112145

**Published:** 2024-10-25

**Authors:** Jiri Volf, Marcela Faldynova, Jitka Matiasovicova, Alena Sebkova, Daniela Karasova, Hana Prikrylova, Hana Havlickova, Ivan Rychlik

**Affiliations:** Veterinary Research Institute, 621 00 Brno, Czech Republic; volf@vri.cz (J.V.); faldynova@vri.cz (M.F.); matiasovicova@vri.cz (J.M.); asebkova@vri.cz (A.S.); karasova@vri.cz (D.K.); prikrylova@vri.cz (H.P.); havlickova@vri.cz (H.H.)

**Keywords:** chicken, caecum, microbiota, probiotics, *Bacteroides*, *Megamonas*, *Megasphaera*

## Abstract

There are extensive differences in the caecal microbiota of chicks from hatcheries and those inoculated with faecal material from adult hens. Besides differences in microbial composition, the latter chickens are highly resistant to *Salmonella* Enteritidis challenges, while the former are susceptible. In this study, we tested whether strains from genera *Bacteroides*, *Megamonas,* or *Megasphaera* can increase chicken resistance to *Salmonella* and *Campylobacter jejuni* when defined microbial mixtures consisting of these bacterial genera are administered. Mixtures consisting of different species and strains from the above-mentioned genera efficiently colonised the chicken caecum and increased chicken resistance to *Salmonella* by a factor of 50. The tested mixtures were even more effective in protecting chickens from *Salmonella* in a seeder model of infection (3–5 log reduction). The tested mixtures partially protected chickens from *C. jejuni* infection, though the effect was lower than that against *Salmonella*. The obtained data represent a first step for the development of a new type of probiotics for poultry.

## 1. Introduction

Chickens represent the most widespread source of animal protein that is acceptable for nearly all communities, and, not surprisingly, the highly efficient commercial production of chickens is practised daily worldwide. However, chickens also represent a reservoir of zoonotic agents like *Salmonella* or *Campylobacter*.

Chickens in commercial production are hatched from disinfected eggs in a clean hatchery environment, despite *Gallus gallus* being evolved to hatch in nests. While chicks raised in contact with adult hens are colonised by chicken-adapted microbiota within the first days of life [1,2,3], the intestinal tract of chicks from hatcheries is colonised randomly by microbiota from environmental sources. The speed of the colonisation of chicks from hatcheries is impossible to predict because of its random nature; however, when averaged over different studies, weeks to months are usually required for chickens to become colonised by chicken-specific gut microbiota [4,5,6,7]. In the meantime, poorly colonised chicks from hatcheries remain susceptible to infections with *Salmonella* or *Campylobacter*, while those colonised by adult-type microbiota are resistant [1,8].

Since the introduction of adult hens to newly hatched chickens is logistically impossible due to the extreme numbers of produced chickens and the risk of the introduction of undesired microbiota into flocks, alternative solutions based on the administration of beneficial microbes have been tested. Unfortunately, probiotics based mostly on Lactobacilli have not proven to be too effective [9], and complex competitive exclusion products, though highly effective in protecting chickens against *Salmonella* infection [10,11], are not generally accepted due to their undefined composition. A compromise between single-species probiotics at one side and undefined complex microbiota on the other side is therefore sought.

In an attempt to identify the most relevant gut microbiota members, we performed multiple experiments in which we determined the composition of the caecal microbiota of one-week-old chicks, either after the oral administration of caecal extracts from adult hens or after one-week-long contact with an adult hen [1,2,12]. Using this approach, we identified bacteria which are usually absent in the gut microbiota of chickens from hatcheries but are readily transferred from hens to offspring. Such gut microbiota members are predominantly characterised as having a strictly anaerobic metabolism, which prohibits them from prolonged survival under aerobic conditions [13,14]. Such characteristics are typical for different representatives belonging to phylum Bacteroidetes, members of class Negativicutes (*Megamonas*, *Megasphaera*, *Veillonella*, or *Phascolarctobacterium*) from Firmicutes, anaerobic Proteobacteria such as *Desulfovibrio*, *Sutterella*, *Parasutterella,* or *Succinatimonas*, and a few other gut anaerobes (e.g., *Cloacibacillus*, *Elusimicrobium*, or *Akkermansia*). A mixture of these taxa increases chicken resistance to colonisation with avian pathogenic *E. coli* [15]; however, whether such bacteria also protect against *Salmonella*, *Campylobacter*, and other pathogens is not known. In this study, we therefore performed multiple independent experiments in which we orally inoculated chicks on their first day of life with different mixtures of chicken gut anaerobes consisting of the above-mentioned taxa, and we tested their protective effect against *Salmonella* and *Campylobacter*. Initially, we aimed to select the most effective strain combination, and this is why the mixtures used in different experiments differed in strain composition. The tested mixtures indeed increased chicken resistance to *Salmonella*, but, rather unexpectedly, the final species and strain selection was irrelevant, as long as the basic principles of combining strains from the above-mentioned taxa were retained.

## 2. Materials and Methods

### 2.1. Experimental Design

**Figure 1:**
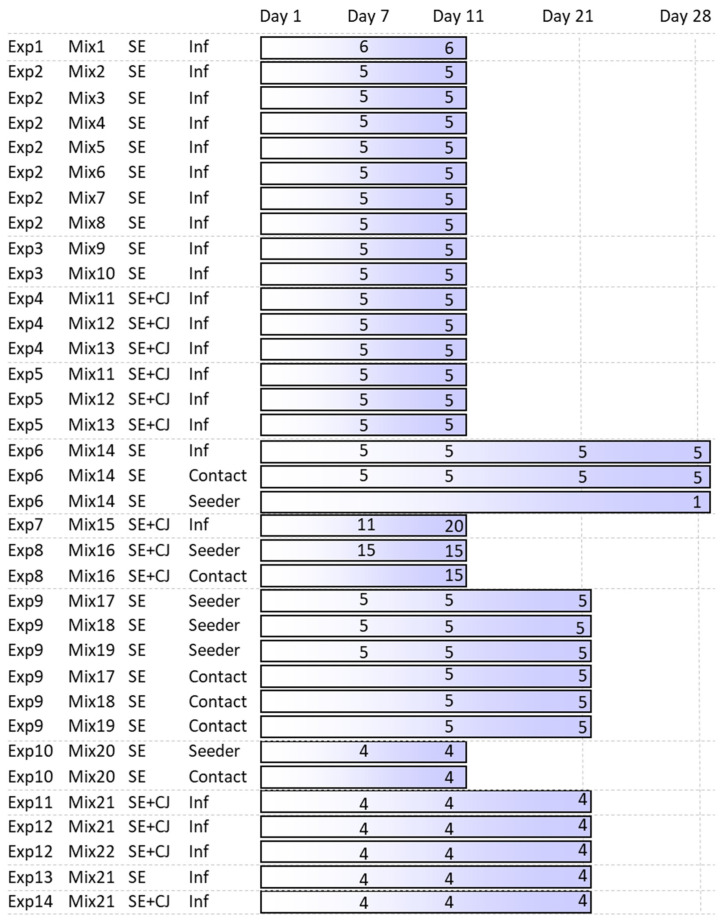
Experimental design of this study. Twenty-two different probiotic mixtures (Mix) have been tested in 14 independent experiments (Exp). Coinfection with *S*. Enteritidis and *C. jejuni* was tested in 7 experiments, and a seeder bird model of infection was used in 4 experiments. Numbers indicate the number of chicks sacrificed at each time point. Control chicks not inoculated with any probiotic mixture were included in each experiment, but these are not shown in the figure. Additional details can be found in Table S2.

Probiotic products have been tested in 14 independent experiments performed gradually from 2018 until 2024. In all experiments, chicks in the experimental groups were orally inoculated with a probiotic mixture on their first day of life while control chickens remained without any treatment. One week later, some of the chicks were sacrificed to check for caecal microbiota composition before infection. The remaining chickens in both the experimental and control groups were orally inoculated with 10^7^ CFU of *Salmonella* Enteritidis 147, resistant to nalidixic acid, in 0.1 mL inoculum. Four days later, the chicks were sacrificed, and *Salmonella* counts were determined in the liver and caecum. This set up was used in all experiments, although the numbers of chickens and probiotic mixtures used differed between experiments (Table S1). In addition, in 6 experiments, chicks were sacrificed also at 14 days post-infection, and a single experiment was extended to 21 days post-infection (Figure 1). In 7 experiments, coinfection with *S*. Enteritidis and *Campylobacter jejuni* was used, and in 4 experiments, only some of the chicks were inoculated with *S*. Enteritidis or *C*. *jejuni*, which acted as seeders to the remaining chicks in the group which were subjected to a natural oro-faecal route of infection. Seeder birds were differentiated from the contact chicks by leg rings. The number of chicks per experimental group and the chicken ages in the different experiments ranged from 3 to 6 (Table S2).

Altogether, 680 chickens were included in this study (Table S2). Of these, 244 chickens were sacrificed when one-week old to check for microbiota composition just before infection by 16S rRNA sequencing. Microbiota composition was determined in 88 control chickens and in 156 chickens inoculated with tested probiotic mixtures. After *Salmonella* infection, samples from 166 chickens from the control groups and 270 chickens from the experimental groups were processed. Since *Salmonella* and *Campylobacter* coinfection was used in only 7 experiments, *Campylobacter* counts were determined only in 84 control and 112 experimental chickens.

Altogether, 52 different strains were tested in at least 1 experiment (Table S1). These strains have been characterised previously [16,17] or cultured recently as part of the continuous interest in the characterisation of chicken gut microbiota. The inclusion criteria included the absence in the gut microbiota of chicks from hatcheries, efficient transfer from hens to chicks [2], and ability to colonise chicken caecum after a single-dose administration [17]. The strains were grown in an anaerobic chamber (10% CO_2_, 5% H_2_, and 85% N_2_ atmosphere at 37 °C for 2 days on a Wilkins–Chalgren anaerobe agar (ThermoFisher Scientific, Waltham, MA, USA). Although the mixtures differed, the unifying principle was that all the mixtures contained multiple Bacteroidetes or Selenomonadales species supplemented with *Bifidobacterium* or strictly anaerobic Proteobacteria, e.g., *Succinatimonas*, *Sutterella*, or *Desulfovibrio*, in some of the experiments (Table S1). Final species and strain selection were affected by availability in our laboratory strain collection, accessed on 20 October 2024 (https://probio.vri.cz/en/catalogue-of-chicken-gut-microbiota-members/). The number of strains in the different mixtures ranged from 3 to 30.

### 2.2. Salmonella and Campylobacter Culture

After the termination of each of the experiments, approx. 0.5 g of caecal content and liver tissue were removed, homogenised in 5 mL peptone water, tenfold serially diluted, and plated on a Xylose Lysine Deoxycholate (XLD) agar supplemented with nalidixic acid. *S*. Enteritidis colonies were counted after 48 h of aerobic incubation at 37 °C. In the case of no *Salmonella* colonies after direct plating, peptone water homogenates were processed according to the ISO 6579 protocol (https://www.iso.org/standard/56712.html, accessed on 20 October 2024) for qualitative *Salmonella* detection. *S*. Enteritidis counts were logarithmically transformed, and samples positive only after the ISO protocol were assigned a value of 1. *Salmonella*-negative samples were given a value of 0. When *Salmonella*–*Campylobacter* coinfection was used, the same dilutions which were used for *Salmonella* enumeration were plated also on a Charcoal cefoperazone deoxycholate agar (CCDA). CCDA plates were then incubated under microaerophillic conditions (CAMPYGEN, ThermoFisher Scientific, Waltham, MA, USA) at 37 °C for 2 days, and *Campylobacter* colonies were counted.

### 2.3. Analysis of Gut Microbiota by Sequencing of V3/V4 Variable Region of 16S rRNA Gene

Microbiota composition was determined as described previously [2]. The samples were homogenised in a MagNALyzer (Roche, Basel, Switzerland). Following homogenisation, the DNA was extracted using a QIAamp DNA Stool Mini Kit (Qiagen, Hilden, Germany) according to the manufacturer’s instructions, and the DNA concentration was determined spectrophotometrically. DNA samples were diluted to 5 ng/mL and were used as templates in the polymerase chain reaction (PCR) with forward primer 5′-TCGTCGGCAGCGTCAGATGTGTATAAGAGACAG-MID-GT CCTACGGGNGGCWGCAG-3′ and reverse primer 5′-GTCTCGTGGGCTCGGAGATGTGTATAAGAGACAG-MID GT GACTACHVGGGTATCTAATCC-3′. The MIDs shown above represent different sequences that were 5, 6, 7, or 9 base pairs in length and that were used to identify individual samples within the sequencing groups. PCR amplification was performed using a HotStarTaq Plus Master Mix kit (Qiagen), and the resulting PCR products were purified using AMPure beads (Beckman Coulter, Brea, CA, USA). In the next step, the concentration of PCR products was determined spectrophotometrically, the DNA was diluted to 100 ng/µL, and groups of 14 PCR products with different MID sequences were indexed with the same indices using the Nextera XT Index Kit (Illumina, San Diego, CA, USA). Prior to sequencing, the concentrations of differently indexed samples were determined using a KAPA Library Quantification Complete kit (Kapa Biosystems, Wilmington, MA, USA). All indexed samples were diluted to 4 ng/µL, and 20 pM phiX DNA was added to a final concentration of 5% (*v*/*v*). Sequencing was performed using a MiSeq Reagent Kit v3 and MiSeq apparatus (Illumina).

Analysis of sequencing data was performed with QIIME 2 [18]. Raw sequence data were demultiplexed and quality-filtered, and sequencing primers were clipped using Je [19] and fastp [20]. The resulting sequences were denoised with DADA2 [21]. Taxonomy was assigned to ASVs using the q2-feature-classifier [22] and classify-sklearn naïve Bayes taxonomy classifier against the Silva 138 [23]. All the software tools were used with default settings.

The colonisation ability of each of the tested strains was extracted from the whole microbiota composition determined by 16S rRNA sequencing. As we knew the whole genomic sequence of each strain including the sequence of the 16S rRNA gene, corresponding ASVs could be identified in the samples of treated chickens. Such ASVs were absent from the microbiota of control chicks as well as the microbiota of experimental chicks treated with mixtures not containing the target strain (see also Table S3).

### 2.4. Statistics

Orally infected chicks including the seeder birds were considered infected and were treated separately from the contact chickens. A *t*-test was used to evaluate the difference in *S*. Enteritidis and *Campylobacter* counts in the caecum and liver of control and experimental chickens. Microbiota abundance in control and experimental chickens was compared by the Mann–Whitney test. In all cases, comparisons with *p* < 0.05 were considered significantly different.

### 2.5. Ethical Statement

The handling of animals in the study was performed in accordance with current Czech legislation (Animal Protection and Welfare Act No. 246/1992 Coll. of the Government of the Czech Republic). The specific experiments were approved by the Ethics Committee of the Veterinary Research Institute, followed by the Committee for Animal Welfare of the Ministry of Agriculture of the Czech Republic (permit number MZe1922 approved on 15 January 2018 and MZe2405 approved on 5 March 2023).

## 3. Results

### 3.1. Colonisation Ability

**Figure 2:**
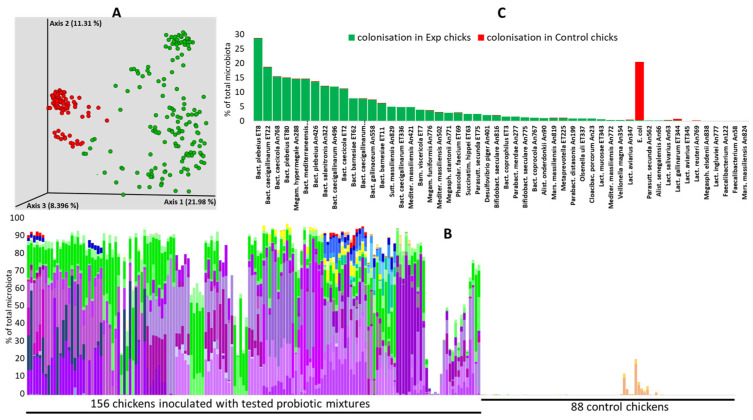
Caecal microbiota of one-week-old chickens. Experimental chickens were orally inoculated by defined mixtures of gut anaerobes on the day of hatching, and, one week later, caecal microbiota composition was determined. Panel (**A**)—used strains efficiently colonised inoculated chickens, which led to the separation of control (red dots) and experimental chickens (green dots) in PCoA analysis using Bray–Curtiss matrix distances. Panel (**B**)—tested strains were absent from the microbiota of control chickens but commonly formed around 80% of the total microbiota in experimental chickens. Shades of purple—different Bacteroidetes isolates (*Bacteroides*, *Barnesiella*, *Parabacteroides*, *Alistipes*, *Mediterranea,* or *Marseilla*), shades of green—Selenomonadales (*Megamonas*, *Megasphaera*, *Phascolarctobacterium*, or *Veillonella*), shades of yellow—Actinobacteria (*Bifidobacterium* or *Olsenella*), shades of blue—Proteobacteria (*Desulfovibrio*, *Sutterella*, *Parasutterella*, or *Succinatimonas*), red—*Cloacibacillus*. Shades of brown in control chickens (*Lactobacillus*). *E. coli*, common in the control chickens, has not been included in the panel (**B**). For additional details, see Table S3. Panel (**C**)—the most efficient colonisers included different *Bacteroides* species, *Barnesiella*, *Megamonas,* and *Sutterella,* which all formed more than 3% of the total microbiota one week after their administration but were absent from the microbiota of control chickens.

Of all tested strains, only two *Faecalibacterium* isolates and *Marseilla massilliensis* An824 did not colonise the chicken caecum one week after inoculation. All the remaining strains colonised the chicken caecum, and, consequently, PCoA clustering separated the chicks from the experimental groups and the control chicks (Figure 2A). The provided probiotic strains altogether formed 78% of the total caecal microbiota in one-week-old chicks on average, and, except for *E. coli* and Lactobacilli, the used strains were absent from the microbiota of control chicks (Figure 2B, Table S3). Different *Bacteroides* species, *Mediterranea*, *Barnesiella*, *Megamonas*, and *Sutterella*, belonged to the genera which colonised the chicken caecum the most since all these isolates each formed more than 3% of the caecal microbiota on day 7 of life, just prior to challenges with *S*. Enteritidis. On the other hand, all tested Lactobacilli were poor colonisers. Namely, *L. mucosae* formed 0.6% of the total microbiota, and the remaining six tested species formed less than 0.5% of the total caecal microbiota one week after their experimental administration (Figure 2C).

### 3.2. The Protective Effect of Defined Bacterial Mixtures Against Salmonella Enteritidis Challenges

**Figure 3:**
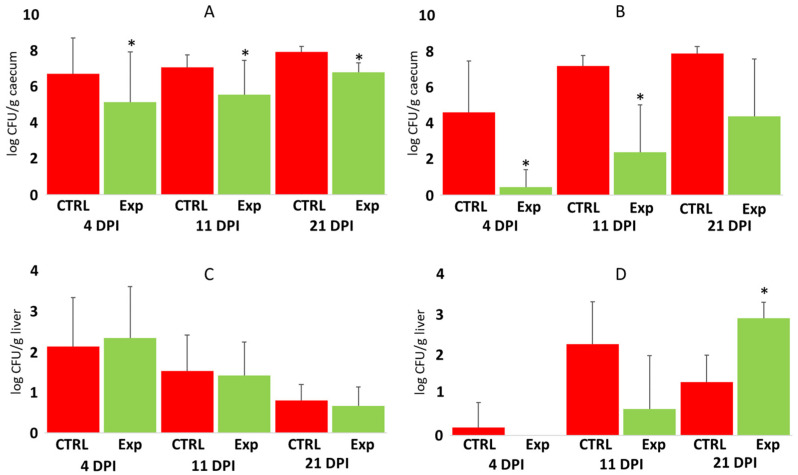
*Salmonella* counts in the caecum and liver of control and probiotic-treated chickens. Panel (**A**)—*Salmonella* counts in the caecum of orally challenged chickens. Panel (**B**)—*Salmonella* counts in the caecum of chickens in contact with the orally challenged seeder chickens. Panel (**C**)—*Salmonella* counts in the liver of orally challenged chickens. Panel (**D**)—*Salmonella* counts in the liver of chickens in contact with seeder chickens. Red columns—*Salmonella* counts in control chickens; green columns—*Salmonella* counts in experimental chickens treated with probiotic mixtures on day 1 of their life. DPI, days post-infection. *—*p* < 0.05 to appropriate control group. For control chicks, n = 120 at 4 DPI, n = 36 at 11 DPI, and n = 10 at 21 DPI. For infected chicks in panels (**A**,**C**), n = 160 at 4 DPI, n = 40 at 11 DPI, and n = 6 at 21 DPI. For contact chicks in panels (**B**,**D**), n = 39 at 4 DPI, n = 20 at 11 DPI, and n = 5 at 21 DPI.

The administration of probiotic mixtures significantly increased chicken resistance to *S.* Enteritidis colonisation in the caecum 4, 11, and 21 days post-infection. In the chickens infected directly by oral gavage, probiotic mixtures increased their resistance by approx. a factor of 50 (Figure 3A). The protective effect of probiotic administration was expressed more in the contact chickens since probiotic mixture-treated contact chickens were 1000–100,000 times more resistant to *Salmonella* caecum colonisation than control, probiotic-untreated chickens (Figure 3B).

Unlike caecum colonisation, probiotic pretreatment did not result in increased resistance to *S.* Enteritidis dissemination and multiplication in the liver. *S.* Enteritidis counts in the liver were similar in the control and experimental chickens, both in the chickens directly inoculated with *S.* Enteritidis, and in the contact chickens (Figure 3C,D).

### 3.3. Performance of Probiotic Mixtures in Individual Experiments

**Figure 4:**
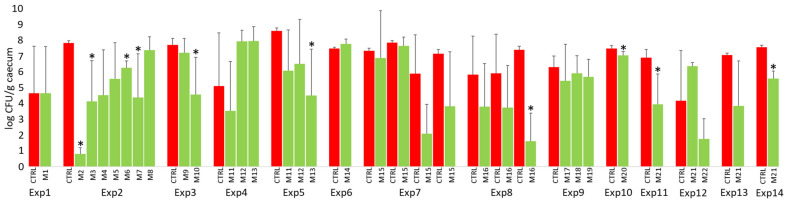
*Salmonella* counts in the caecum of control and probiotic-treated chickens in individual experiments at 4 days post-infection. Significant protection 4 days post-infection was recorded only in 10 cases, both due to the low number of chicks used in individual experiments (n = 3–6). In addition, in some experiments, e.g., in experiments 1, 4, or 12, the chicks in the control group were of naturally increased resistance to *Salmonella* challenges. Red columns—*Salmonella* counts in control chickens, green columns—*Salmonella* counts in the chickens treated with probiotic mixtures. Asterisks indicate a significant difference to the control group from the same experiment. For the composition of tested mixtures and the number of chicks used in each experiment, see Tables S1 and S2.

As shown in Figure 3, probiotic mixtures protected chickens against *S*. Enteritidis. However, this does not mean that the protective effect was recorded repeatedly in all the experiments. When individual experiments were evaluated, the significant protection in directly challenged chickens 4 days post-infection was recorded only in 10 cases. The significance did not follow any logic as, for example, in experiment 2, mixture M2 contained seven bacterial species (*Bacteroides caecicola* An768, *Bacteroides caecigallinarum* An496, *Bacteroides salanitronis* An322, *Mediterranea massiliensis* An421, *Megamonas funiformis* An776, *Megamonas hypermegale* An288, and *Megasphaera elsdenii* An771) and mixtures M3-M8 contained one additional bacterial strain in addition to the seven core species (see Table S1 for the composition of all tested mixtures). Similarly, in experiments 4 and 5, three identical mixtures were tested repeatedly: mixture M11 contained eight *Bacteroides* species; mixture M12 consisted of three Veillonellaceae species; and mixture M13 consisted of both *Bacteroides* and Veillonellaceae species (Table S1). An additive effect resulting in significant protection was recorded in experiment 5, while in experiment 4, *Salmonella* counts in the caecum of chickens treated with a mixture of *Bacteroides* and Veillonellaceae were numerically higher than in the control chickens (Figure 4). Exactly the same mixture M21 was used in the last four experiments, and the protection of this mixture against *Salmonella* challenges reached statistical significance in only two of them (Figure 4).

### 3.4. Protective Effect of Administering Defined Mixtures Against Campylobacter jejuni Challenges

**Figure 5:**
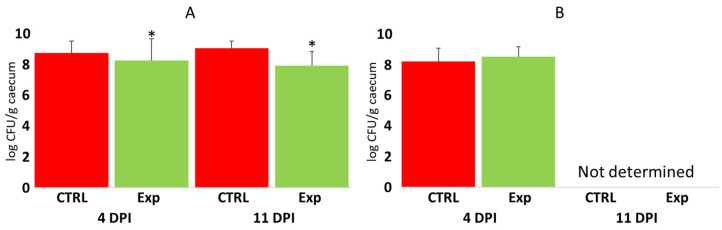
*Campylobacter* counts in the caecum of control and probiotic-treated chickens. Panel (**A**)—*Campylobacter* counts in the caecum of directly challenged chickens. Panel (**B**)—*Campylobacter* counts in the caecum of chickens in contact with directly challenged seeder chickens. Red columns—*Campylobacter* counts in control chickens; green columns—*Campylobacter* counts in the chickens treated with probiotic mixtures. DPI, days post-infection. Asterisk indicates significant difference to control group. For control chicks, n = 72 at 4 DPI and n = 12 at 11 DPI. For infected chicks in panel A, n = 81 at 4 DPI and n = 16 at 11 DPI. For contact chicks in panel B, n = 15 at 4 DPI.

The protective effect of the tested probiotic mixtures against *Campylobacter* was measured only 4 and 11 days post-infection in the case of directly challenged chickens, and only 4 days post-infection in contact chickens. *Campylobacter* colonisation resulted in bacterial counts that were 10 times higher that of *Salmonella* (compare Figure 3A,B with Figure 5A,B). *Campylobacter* therefore exhibited a different mode of caecum colonisation compared to *Salmonella*. This difference was further confirmed by the low, though significant, protective effect of administered probiotics in directly challenged chickens (Figure 5).

### 3.5. Protective Effect of Administration of Defined Mixtures Against Opportunistic Pathogens

**Figure 6:**
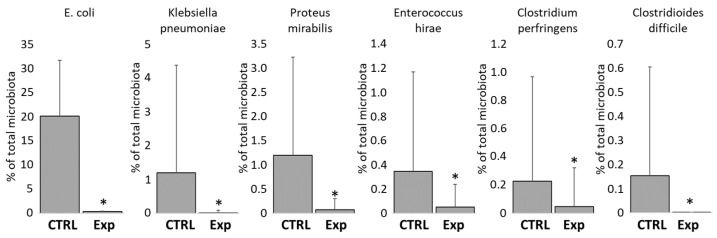
Protective effect of tested probiotic mixtures against opportunistic pathogens of environmental origin. Analysis of 16S rRNA sequencing data in one-week-old chickens showed that the provided probiotic mixtures significantly decreased the abundance of *E. coli*, *Klebsiella pneumoniae*, *Proteus mirabilis*, *Enterococcus hirae*, *Clostridium perfringens*, and *Clostridioides difficile*. These species were always significantly more abundant in the caecal microbiota of one-week-old control chickens than in those treated with tested probiotic mixtures. Asterisks indicate significant difference. For control chicks, n = 88. For experimental chicks, n = 156.

Finally, we checked whether the provided probiotic mixtures may protect chickens also against opportunistic pathogens. Data from 16S rRNA sequencing (Table S3) showed that the abundance of *E. coli*, *Klebsiella pneumoniae*, *Proteus vulgaris*, *Enterococcus hirae*, *Clostridium perfringens,* and *Clostridiodes difficile* were significantly lower in the caeca of chicks from experimental groups than in the non-treated controls (Figure 6). The administration of defined bacterial mixtures therefore increased the resistance of chickens to colonisation by other bacterial pathogens.

## 4. Discussion

In this study, we tested whether different mixtures of gut anaerobes can limit the colonisation of *S*. Enteritidis and *Campylobacter* in chickens. Species and strains were selected mainly from those which are efficiently transferred through contact between chicks and adult hens [2] and those which colonise the chicken caecum after a single-dose oral administration [3,17]. We therefore did not consider the metabolism of the individual strains tested, though it is known that *Bacteroides* are highly dependent on polysaccharide and carbohydrate fermentation, *Megamonas* sp. are of intermediate dependence on carbohydrate fermentation, and *Phascolarctobacterium* and *Sutterella* are preferentially amino acid degraders [24]. We did not consider how individual mixtures may affect the composition of low molecular weight molecules in the caecal digesta [25] or interact with secretory IgA [26,27]. None of the tested strains encoded flagella, a TLR5 ligand, and none of the tested strains represented spore-forming bacteria [16]. Considering such properties when selecting probiotic mixtures may increase their efficacy in the future.

Despite using rather broadly defined inclusion parameters, the selected mixtures protected chickens against both *Salmonella* and *Campylobacter,* though the protective effect against *Campylobacter* was lower than that against *Salmonella*. No protection of competitive exclusion products against *Campylobacter* has been reported recently [28], confirming our observation that the selection of probiotic mixtures for protection against *Campylobacter* may be a difficult task. We showed earlier that *Campylobacter* was transferred by the oral inoculation of chicks with caecal contents from adult hens, which also means that other co-transferred microbiota members did not prevent *Campylobacter* colonisation [29].

The tested mixtures increased the resistance of chickens to oral infection with *Salmonella* by approximately a factor of 50. More interesting was the effect of the tested mixtures in contact chickens where the protection was in a range of 3–5 logs of magnitude. The use of such products should be mainly preventive to reduce the spread of infection in flocks rather than to consider the protection of individual chickens. Despite significant protection, the efficacy of defined mixtures can be further improved as it was numerically lower in comparison with the use of complex microbiota preparations [1,10,11]. Even washes of bacterial colonies growing on agar plates inoculated by the caecal contents of adult hens were more efficient [2], which indicates that there are species that increase the efficacy of defined products further and that these can be grown in vitro. Whether these should be flagella-expressing bacteria to stimulate TLR5 signalling [30,31], spore formers to mimic the probiotic effect of *Bacillus* spores [32,33], butyrate producers, S-layer protein-expressing bacteria [24], or some other remains to be determined. We also noticed that some but not all *Bacteroides* strains tested in this study (*B. plebeius* An426, *B. barnesiae* ET62, *B. mediterraneensis* An793, *B. caecigallinarum* ET336) encoded genes for type VI secretion systems [34,35]. However, whether this provided them with extra competitiveness and increased chicken resistance to *Salmonella* and *Campylobacter* remains to be determined.

It can be argued that since probiotic mixtures of different compositions were used in individual experiments, these cannot be grouped together and compared with non-inoculated controls. The reason for such an approach is that this study summarises our experience in the selection of novel types of probiotics which can protect chickens against *Salmonella* or *Campylobacter* infection. To select the most effective mixture, we therefore combined and tested different species and sometimes even different isolates belonging to the same species. Unlike the original expectation that mixtures of different composition will exhibit different ranges of protection, we concluded that if using *Bacteroides*, *Barnesiella*, *Megamonas*, *Megasphaera*, *Phascolarctobacterium*, *Succinatimonas*, or *Sutterella* without any additional criteria applied, the final species and strain selection is not critically important for protection from *Salmonella*. Host adaptation should be respected [13,14], and if we also consider the poorer growth of *Barnesiella*, *Phascolarctobacterium*, or *Sutterella* in vitro, mixtures should contain a variety of species from Bacteroidaceae and Veillonellaceae since it has been shown that colonisation with individual strains does not lead to any protection [17]. Although it cannot be excluded that there might be isolates with unusual extra probiotic characteristics, such cases seem to be rare. It is therefore more appropriate to expect that if mixtures of around 10 strains are prepared from representatives of Bacteroidaceae and Veillonellaceae, a similar protective effect, as described in this study, can be expected, irrespective of the selected species and strains.

## Data Availability

The data supporting the conclusions of this article are included within the article. Additional data used and/or analysed during the current study are available from the corresponding author upon reasonable request.

## Supplementary Materials

The following supporting information can be downloaded at https://www.mdpi.com/article/10.3390/microorganisms12112145/s1, Table S1. List of strains and mixtures tested in at least one experiment of the whole study; Table S2. Number of chickens used in individual experiments of this study; Table S3. ASV (amplicon sequence variants) table of all strains recorded in this study.
